# The efficacy of ultrasonic ablation combined with medication in the treatment of adenomyosis: a systematic review and network meta-analysis

**DOI:** 10.3389/fphar.2026.1767202

**Published:** 2026-06-12

**Authors:** Qi Qin, Ruiqi Wang, Yu Yao, Lili Zhang, Shuyue Pang, Xiuxiang Zhang, Rui Ma, Yanping Wang

**Affiliations:** 1 College of Traditional Chinese Medicine, Changchun University of Chinese Medicine, Changchun, China; 2 Department of Obstetrics and Gynecology, Affiliated Hospital of Liaoning University of Traditional Chinese Medicine, Shenyang, China; 3 The Affiliated Hospital of Changchun University of Traditional Chinese Medicine, Changchun, China; 4 Obstetrics and Gynecology Diagnosis and Treatment Center, The Affiliated Hospital of Changchun University of Traditional Chinese Medicine, Changchun, China; 5 Research Center of Traditional Chinese Medicine, The Affiliated Hospital of Changchun University of Traditional Chinese Medicine, Changchun, China

**Keywords:** adenomyosis, combined therapies, high-intensity focused ultrasound, medication, network meta-analysis, systematic review

## Abstract

**Background:**

Adenomyosis occurs when tissue from the inner lining of the endometrium grows into the muscle wall of the myometrium. Previous studies have suggested that High-Intensity Focused Ultrasound (HIFU) combined with medications may improve treatment outcomes for adenomyosis; however, the comparative efficacy of different medication combinations remains unclear. This network meta-analysis aimed to compare and rank the efficacy of various medications combined with HIFU in patients with adenomyosis.

**Methods:**

Randomized controlled trials reporting on HIFU combined with medications for adenomyosis were retrieved from 8 databases from their inception to 23 June 2025. The literature meeting the inclusion criteria was evaluated for quality and risk of bias using the Cochrane 5.1 manual and RoB2. Subsequently, a Bayesian network meta-analysis was conducted using R software.

**Results:**

A total of six interventions (HIFU + GnRH-a, HIFU + testosterone propionate, HIFU + percutaneous ethanol injection, HIFU + microbubble contrast agent, HIFU + oxytocin, and HIFU + microbubble contrast agent + oxytocin) were included to evaluate their efficacy on intraoperative indicators of adenomyosis compared with HIFU alone. A total of 8,700 records were retrieved, with 16 studies ultimately included, involving 1,685 patients with adenomyosis. Compared with HIFU alone, HIFU combined with ethanol ablation demonstrated the highest efficacy in non-perfused volume ratios (NPVR) (Mean Difference (MD) = 27, 95% Confidence Interval (CI): 2.7 to 52), sonication energy (MD = −230%, 95% CI: −290 to −180), and treatment duration (MD = −44%, 95% CI: −80 to −7.8); Additionally, HIFU combined with Gonadotropin-releasing hormone agonist (GnRH-a) showed superiority in energy efficiency factor (MD = −31%, 95% CI: −90 to −13).

**Conclusion:**

The efficacy of HIFU combined with medications in the treatment of adenomyosis is superior to that of HIFU alone. Different medications offer distinct advantages in enhancing the therapeutic effect of HIFU.

**Systematic Review Registration:**

clinicaltrials.gov, identifier CRD420251143604.

## Introduction

Adenomyosis refers to the penetration of endometrial glands and stroma into the myometrium, manifesting clinically as increasingly severe dysmenorrhea, infertility, and intense menstrual bleeding, among others. The pain associated with the disease and anxiety about pregnancy significantly impact the daily lives of patients. Adenomyosis primarily occurs in women of childbearing age, with approximately 20% of patients being under 40 years old and 80% being between 40 and 50 years old (Harada et al., 2016). Epidemiological research (Naftalin et al., 2012), revealed that adenomyosis occurs in 20.9% of cases, with prevalence escalating with age, reaching a high of 32% in women aged 40–49 years. Despite adenomyosis being a non-malignant condition, approximately 18.0% of individuals concurrently suffer from endometriosis, and nearly 47.6% exhibit uterine fibroids, which may be accompanied by endometrial cancer (Yu et al., 2020). Therefore, investigating effective feasible therapeutic approaches in the early stages of adenomyosis is crucial.

Current treatments for adenomyosis include both medical and surgical options. Hormonal or non-hormonal medications are the primary therapeutic choice. If medication proves ineffective, surgical intervention may be considered. While excising lesion allows for its complete removal in a single procedure, recurrence rates remain as high as 19% (Younes and Tulandi, 2018). Hysterectomy provides curative results but is unsuitable for patients wishing to preserve their uterus (Capezzuoli et al., 2024). High-Intensity Focused Ultrasound (HIFU) is currently a widely adopted uterus-preserving treatment in clinical practice, demonstrating proven efficacy (Jeng et al., 2020; Chen et al., 2024; Younes and Tulandi, 2018). It is non-invasive and safe, operating on the principle that low-energy ultrasound waves are focused through a specific probe, concentrating energy within the adenomyotic lesion area of the uterus to create a high-temperature therapeutic point measuring only a few millimeters in diameter, thus inducing necrosis of the lesion. Moreover, the coagulative necrosis caused by HIFU results in significantly less pain compared to the ischemic necrosis induced by uterine artery embolization (Dueholm, 2018). As a non-invasive surgical therapy, HIFU is increasingly favored by patients wishing to preserve fertility due to its minimal trauma, low complication rate, reduced hospital stay, and lack of impact on daily life (Dong and Yang, 2010). Research (Bahutair and Alhubaishi, 2024) indicates that quality of life improvements of 50%–80% can be achieved within 3–12 months following HIFU treatment. However, current HIFU remains limited by suboptimal NPVR, pronounced pain-related side effects, and patient intolerance during procedures.

Consequently, increasing clinical research is exploring the combined use of HIFU with pharmaceutical agents to investigate their clinical efficacy and safety during and after treatment. Examples include HIFU combined with GnRH-a and HIFU combined with oxytocin. Oxytocin is a multifunctional peptide hormone that binds to specific oxytocin receptors (OXTR) on the uterine myometrium, stimulating uterine smooth muscle contractions (Hermesch et al., 2024). This action compresses blood vessels within the myometrium and lesion, significantly reducing blood flow and heat dissipation. As a result, HIFU can focus more effectively on the lesion, thereby enhancing the treatment efficacy (Carter et al., 2020). A clinical study (Zhang demonstrated that oxytocin application during HIFU significantly reduced the energy required for HIFU ablation of adenomyosis without adverse effects. Pre-treatment with pure ethanol (PEI) prior to HIFU application may substantially lower the acoustic power threshold needed for HIFU-induced inertial cavitation and accelerate tissue temperature rise at lower power levels. Concurrently, ethanol evaporation dissipates substantial heat through an ‘evaporative cooling’ effect, preventing tissue overheating beyond water’s boiling point and potentially reducing adverse effects (Chen et al., 2012). In studies combining HIFU with PEI for uterine fibroid treatment (Zhang et al., 2022), ultrasound observations following PEI injection into lesions revealed complete vascular occlusion at the maximum volume, indicating that PEI improves the acoustic environment for ultrasound ablation. GnRH-a represents a potent hormonal therapy commonly employed for adenomyosis, encompassing agents such as leuprolide, goserelin, and triptorelin. It rapidly reduces uterine volume and significantly alleviates dysmenorrhea and menorrhagia, though its effects are transient and reversible. When combined with GnRH-a, HIFU demonstrates more pronounced effects in reducing both uterine and lesion volumes (Pang et al., 2021; Peng et al., 2021; Grow and Filer, 1991). While various combination therapies possess distinct advantages, the optimal combination approach for treating adenomyosis using HIFU alongside pharmacological agents remains unclear in terms of intraoperative and postoperative efficacy.

This systematic review and network meta-analysis aims to investigate the efficacy and safety of ultrasound ablation combined with various drugs, compared to ultrasound ablation alone, in the treatment of adenomyosis. It includes randomized controlled trials of HIFU combined with drug therapy.

Objective 1: To synthesize existing clinical protocols for HIFU combined with drug therapy in the treatment of adenomyosis.
Objective 2: To assess the efficacy of different treatment approaches in relation to intraoperative and postoperative adverse reactions.
Objective 3: To identify the optimal treatment regimen for HIFU combined with drug therapy in adenomyosis, thereby providing data to support its clinical application.

## Materials and methods

A systematic search was conducted in PubMed, Web of Science, Cochrane, Embase, China National Knowledge Infrastructure Database (CNKI), Wanfang Database, VIP Database, and China Biology Medicine Database (CBM) until 23 June 2025. The study protocol was developed following the Preferred Reporting Items for Systematic Reviews and Meta-Analyses (PRISMA) guidelines and the Cochrane Handbook, and it was registered in PROSPERO (CRD420251143604). This systematic review is reported in accordance with the PRISMA-NMA statement. The PRISMA checklist is provided in Supplementary Table S1. The Version 2 of the Cochrane risk-of-bias tool for randomized trials (RoB2) was utilized to assess the risk of bias for each included study. Each included trial was assessed based on five domains: randomization process, deviation from the intended intervention, incomplete outcome data, bias in outcome assessment, and selective reporting of outcomes. Each trial was rated “low risk,” “high risk,” or “unclear risk.” Risk of bias was assessed independently by two authors (Qi Qin and Shuyue Pang). For each disagreement, a discussion was held with a third author (Xiuxiang Zhang) until reaching a consensus.

### Literature screening and data extraction

The basic information of retrieved articles was collected, including authors, year of publication, country/region, study type, interventions, injection volume/frequency/concentration, number of subjects, patient age, body mass index (kg/m^2^), and HIFU manufacturer. Outcome data included ablation rate, energy efficiency factor, irradiation energy, irradiation duration, therapeutic power, treatment duration, time to clearance per unit volume, grayscale changes in target area, and adverse reactions (sacral pain, pain at treatment site, radiation pain, leg pain, lower abdominal pain, and fever). The articles were reviewed, categorized, and screened independently by two authors (Qi Qin and Shuyue Pang), with basic information and relevant data collected. For each disagreement, a discussion was held with a third author (Xiuxiang Zhang) until reaching a consensus.

### Eligibility criteria

The inclusion criteria were constructed based on the PICOS standard:

1. Participants: Patients diagnosed with adenomyosis based on clinical symptoms, laboratory tests, ultrasound, or MRI.
2. Interventions: HIFU-related adjuvant medications
3. Comparison: HIFU alone.
4. Outcomes: Main outcome: NPVR and energy efficiency factor; secondary outcomes: sonication energy, irradiation time, treatment power, treatment duration, time to eliminate per unit volume, presence of grey-scale changes in the target area, and adverse reactions.
5. Research Type: Randomized controlled trials (RCTs).
6. Language: Articles in English or Chinese.

### Exclusion criteria

1. Studies that do not meet the topic requirements.
2. Studies where the sample size of each group was less than 20 cases.
3. Studies that cannot provide complete outcome indicators, where the full text cannot be obtained, or where the statistical methods are incorrect.

### Statistical analysis

For cases where units were inconsistent across studies, we standardized Treatment Power and Sonication time for ablating 1 mm^3^ of lesion to J and s, respectively. The network meta-analysis (NMA) will be conducted using Bayesian methods via the Gemtc package in R software. For continuous outcome variables, the standardized mean difference (MD) with 95% credible intervals (CrI) will be used to pool effect sizes. For binary variables, the risk ratio (RR) with 95% CrI will be applied for effect size combination. Network relationship diagrams will be plotted to provide a simplified summary of the available evidence among various interventions. The overall heterogeneity statistic I^2^ will be calculated. If the overall heterogeneity is high (I^2^ > 50%), a random-effects model will be used; otherwise, a fixed-effect model will be applied for the NMA. If closed loops exist in the network diagram, the node-splitting method will be used to test local inconsistency within the loop; if the test yields P > 0.05, good consistency is considered to exist for that local comparison. League tables will display the results of pairwise comparisons between different interventions. The effectiveness of each intervention will be presented through the surface under the cumulative ranking curve (SUCRA), where a larger SUCRA indicates a higher probability of the intervention being the most effective.

### Publication bias

For results that were statistically significant, both qualitative and quantitative assessments of publication bias were performed using funnel plots in combination with Egger’s test, the Begg-Mazumdar test, and the Thompson test. Specifically, publication bias was assessed using funnel plots and Egger’s linear regression test. For outcomes with at least 10 included studies, Egger’s test was conducted to quantitatively evaluate funnel plot asymmetry, with a two-tailed P value < 0.05 indicating significant publication bias. For outcomes with fewer than 10 studies, only qualitative visual inspection of funnel plots was carried out, as the statistical power of Egger’s test is limited under such conditions.

## Results

### Search results and characteristics of the included studies

**FIGURE 1:**
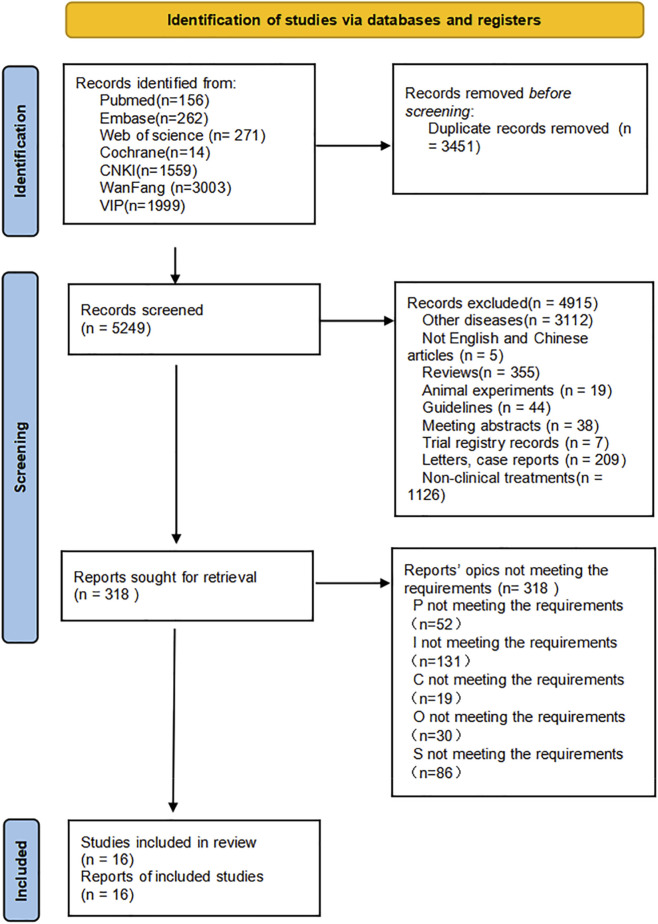
PRISMA flowchart of study selection.

A total of 8,700 articles were retrieved from the database search. We eliminated 3,451 duplicate articles using EndNote. After a careful assessment of titles and abstracts, we excluded 4,915 articles that did not meet the inclusion criteria. Ultimately, 16 randomized controlled trials (RCT) were included in the network meta-analysis. The literature retrieval and screening processes were conducted in accordance with PRISMA guidelines, as illustrated in Figure 1.

The final analysis included 16 studies (Cao Zhengjun, 2017; Chen Meiqin, 2023; Ding Guisheng, 2019; Jia Xiao-Ping, 2023; Li Fengli, 2021; Lingxian, 2016; Ruihong et al., 2021; Song Xuewei, 2024; Wang Yi, 2020; Wu Na, 2022; Wu Xiangrong, 2016; Zhang et al., 2014; Zhang Ting, 2021; Zhang Xin, 2013; Zhang Xin, 2012; Xiaoli, 2018) involving 1,685 patients, who were randomly assigned to either the treatment or control group. The number of participants in each RCT ranged from 66 to 330. All included patients underwent high-intensity focused ultrasound (HIFU) as the primary treatment modality. Six types of interventions were examined: Oxytocin + HIFU (6 articles), Percutaneous Ethanol Injection (PEI) + HIFU (2 articles), GnRH-a + HIFU (5 articles), microbubble contrast agent + HIFU (3 articles), testosterone propionate + HIFU (1 article), and microbubble contrast agent + oxytocin + HIFU (1 article). The basic features of the included studies are summarized in Table 1.

**TABLE 1:** Baseline information and outcome indicators.

Author	Year	Country	Group	n	Age (year)
Song Xuewei (2024)	2024	China	HIFU	60	38.55 ± 5.45
			HIFU + GnRH-a	60	38.78 ± 4.98
Jia Xiao-Ping (2023)	2023	China	HIFU	50	42.54 ± 4.43
			HIFU + GnRH-a	50	42.18 ± 5.61
Cao Zhengjun (2017)	2017	China	HIFU	22	40.27 ± 5.11
			HIFU + GnRH-a	23	41.35 ± 5.76
			HIFU + GnRH-a	21	40.19 ± 5.73
Lingxian (2016)	2016	China	HIFU	47	43.2 ± 7.8
			HIFU + Testosterone Propionate	47	42.5 ± 8.3
Wu Na (2022)	2022	China	HIFU	34	44.7 ± 4.7
			HIFU + GnRH-a	34	44.2 ± 4.6
Ding Guisheng (2019)	2019	China	HIFU	31	-
			HIFU + PEI	47	
Zhang Ting (2021)	2021	China	HIFU	30	-
			HIFU + GnRH-a	30	
			HIFU + GnRH-a	30	
Wang Yi (2020)	2020	China	HIFU	35	40.06 ± 6.21
			HIFU + Microbubble Contrast Agent	35	41.17 ± 5.53
Zhang Xin (2013)	2013	China	HIFU	43	41.0 ± 5.4
			HIFU + Oxytocin	43	41.2 ± 3.9
			HIFU + Oxytocin	43	41.9 ± 4.1
Wu Xiangrong (2016)	2016	China	HIFU	48	-
			HIFU + Oxytocin	48	
Zhang Xin (2012)	2012	China	HIFU	43	-
			HIFU + Oxytocin	43	
Chen Meiqin (2023)	2023	China	HIFU	40	35.17 ± 5.26
			HIFU + Microbubble Contrast Agent	40	35.49 ± 5.63
Li Fengli (2021)	2021	China	HIFU	52	43.37 ± 8.14
			HIFU + PEI	52	43.12 ± 8.05
Xiaoli (2018)	2018	China	HIFU	44	43.73 ± 5.13
			HIFU + Oxytocin	44	43.19 ± 5.26
Ruihong et al. (2021)	2021	China	HIFU + Microbubble Contrast Agent + Oxytocin	82	38.1 ± 6.9
			HIFU + Oxytocin	85	37.0 ± 5.9
			HIFU + Microbubble Contrast Agent	81	36.9 ± 3.8
			HIFU	82	37.9 ± 6.1
Zhang et al. (2014)	2014	China	HIFU	43	41.0 ± 5.4
			HIFU + Oxytocin	43	41.2 ± 3.9

We recorded eight outcome measures: NPVR, energy efficiency factor, sonication energy, irradiation duration, treatment power, treatment duration, ablation per unit volume time, and changes in target area grayscale. Additionally, six adverse reactions were documented: sacral pain, treatment area pain, radiation pain, leg pain, lower abdominal pain, and fever. Detailed baseline information, outcome measures, and adverse reactions are presented in Table 2.

**TABLE 2:** Adverse reaction information.

Author	Year	Sacral pain [N (%)]	Pain in the treatment area [N (%)]	Radiating pain [N (%)]	Leg pain [N (%)]	Lower abdominal pain [N (%)]	Fever [N (%)]
Song Xuewei (2024)	2024	-	-	-	-	-	-
Jia Xiao-Ping (2023)	2023	31 (62.0)	-	10 (20.0)	-	-	-
		29 (58.0)		7 (14.0)			
Cao Zhengjun (2017)	2017	-	-	-	-	-	-
Lingxian (2016)	2016	-	-	-	-	-	-
Wu Na (2022)	2022	16 (47.1)	29 (85.3)	-	-	-	-
		14 (41.2)	30 (88.2)				
Ding Guisheng (2019)	2019	4 (12.9)	-	-	-	-	-
		9 (19.2)					
Zhang Ting (2021)	2021	-	-	-	-	-	-
Wang Yi (2020)	2020	2 (5.7)	-	-	-	4 (11.4)	-
		0 (0)				1 (3)	
Zhang Xin (2013)	2013	26 (60.4)	29 (67.4)	6 (13.9)	-	-	0 (0)
		26 (60.4)	3 (76.7)	2 (4.6)			0 (0)
		26 (60.4)	35 (81.3)	4 (9.3)			0 (0)
Wu Xiangrong (2016)	2016	-	-	-	-	-	-
Zhang Xin (2012)	2012	26 (60.4)	29 (67.4)	6 (13.9)	-	-	0 (0)
		26 (60.4)	33 (76.7)	2 (4.6)			0 (0)
Chen Meiqin (2023)	2023	4 (10.0)	32 (80.0)	-	3 (7.5)	10 (25.0)	3 (7.5)
		1 (2.5)	21 (52.5)		2 (5.0)	8 (20.0)	2 (5.0)
Li Fengli (2021)	2021	3 (5.8)	-	-	-	2 (3.9)	3 (5.8)
		3 (5.8)				5 (9.6)	4 (7.7)
Xiaoli (2018)	2018	-	-	-	-	-	-
Ruihong et al. (2021)	2021	12 (14.6)	-	-	21 (25.6)	69 (84.1)	0 (0)
		10 (11.8)			16 (18.8)	60 (70.6)	1 (1.2)
		11 (13.6)			18 (22.2)	65 (80.2)	0 (0)
		9 (11.0)			17 (20.7)	61 (74.4)	1 (1.2)
Zhang et al. (2014)	2014	-	-	-	6 (13.9)	29 (67.4)	-
					2 (4.6)	33 (76.7)	

### Risk of bias of included studies

**FIGURE 2:**
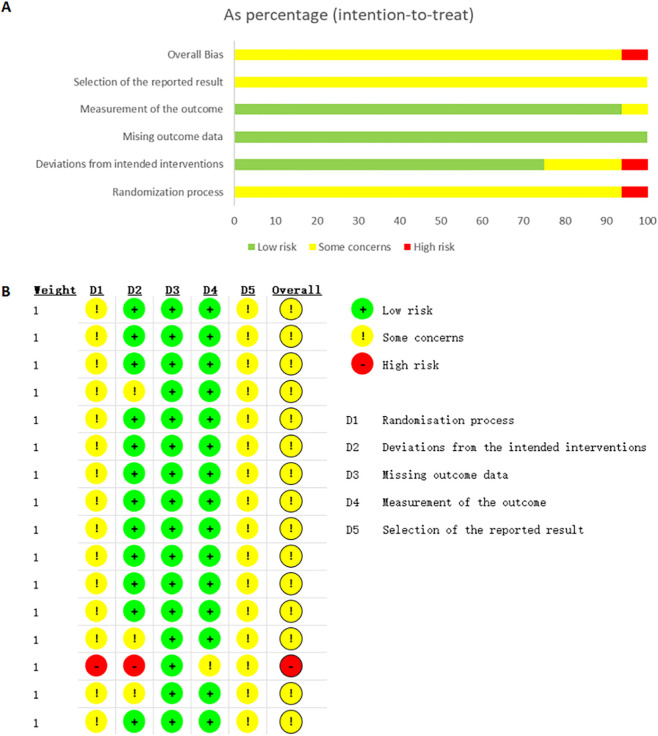
The results of risk of bias. **(A)** Risk of bias graph; **(B)** risk of bias summary.

The risk of bias assessment for each study is shown in Figure 2. Among the 16 included studies, 15 (Song Xuewei, 2024; Chen Meiqin, 2023; Jia Xiao-Ping, 2023; Wu Na, 2022; Li Fengli, 2021; Ruihong et al., 2021; Zhang Ting, 2021; Wang Yi, 2020; Ding Guisheng, 2019; Cao Zhengjun, 2017; Lingxian, 2016; Wu Xiangrong, 2016; Zhang et al., 2014; Zhang Xin, 2013; Zhang Xin, 2012) exhibited a risk of bias of “some concern” across all domains. This was due to the lack of descriptions regarding the knowledge status of intervention implementers in these studies. The remaining study (Xiaoli was rated as having a “high risk” across multiple domains due to the absence of information regarding randomization and whether participants and intervention providers were aware of allocation.

### Primary outcome

#### NPVR

**FIGURE 3:**
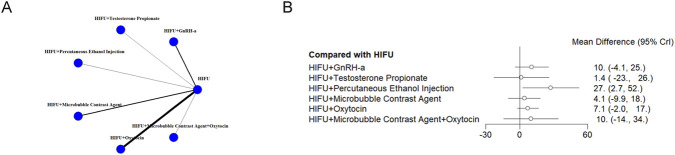
A network meta-analysis of NPVR. **(A)** Network diagram of NPVR; **(B)** forest plot of NPVR. NPVR, non-perfused volume ratios.

A total of 12 studies (Chen Meiqin, 2023; Jia Xiao-Ping, 2023; Zhang Ting, 2021; Wang Yi, 2020; Ding Guisheng, 2019; Xiaoli, 2018; Cao Zhengjun, 2017; Lingxian, 2016; Wu Xiangrong, 2016; Zhang et al., 2014; Zhang Xin, 2013; Zhang Xin, 2012) involving 1,491 patients reported the NPVR for seven treatment modalities (Figure 3A). Ranking probability analysis indicated that among the seven treatment groups, HIFU combined with PEI demonstrated the highest NPVR (SUCRA = 92.7%, I^2^ = 3%). The following combinations exhibited higher NPVR than HIFU alone: HIFU combined with GnRH-a (MD = 10%, 95% CI: −4.1 to 25), HIFU combined with testosterone propionate (MD = 1.4%, 95% CI: −23 to 26), HIFU combined with ethanol (MD = 27%, 95% CI: 2.7 to 52), HIFU combined with a microbubble contrast agent (MD = 4.1%, 95% CI: −9.9 to 18), HIFU combined with oxytocin (MD = 7.1%, 95% CI: −2.10 to 17), and HIFU combined with a microbubble contrast agent plus oxytocin (MD = 10%, 95% CI: −14 to 34) (Figure 3B). Ranking probability analysis indicated that HIFU combined with PEI achieved the highest NPVR among the 7 treatments (SUCRA = 92.7%, I^2^ = 3%; Table 3). Although the results showed that PEI ranked highest in terms of NPVR, its confidence interval was wide and close to zero, indicating marginal statistical significance. Results of pairwise comparisons are presented in Supplementary Table S2.

**TABLE 3:** I^2^ and SUCRA values of outcome measures.

Outcome measures	I^2^ (%)	SUCRA (%)						
		HIFU	HIFU + GnRH-a	HIFU + testosterone propionate	HIFU + percutaneous ethanol injection	HIFU + microbubble contrast agent	HIFU + oxytocin	HIFU + microbubble contrast agent + oxytocin
NPVR	3	17.39	61.85	31.47	92.65	38.26	51.48	56.90
EEF	11	89.81	0.67	-	69.28	63.90	37.12	39.23
Sonication energy	47	44.78	19.73	-	100.00	-	35.49	-
Sonication time	3	16.25	62.44	18.50	57.76	61.86	38.91	94.27
Sonication power	5	30.66	71.16	-	-	52.96	31.48	63.73
Treatment time	3	11.48	57.84	22.96	90.07	80.85	36.81	-
Sonication time for ablating 1 mm^3^ of lesion	37	7.78	84.37	-	-	-	57.84	-
Grayscale changes in target area	0	8.60	43.82	97.58	-	-	-	-

#### Efficacy factors

**FIGURE 4:**
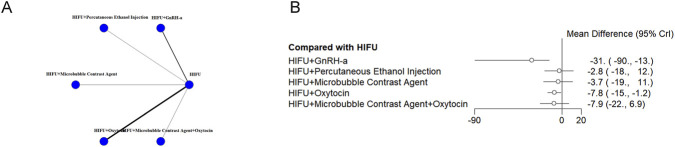
A network meta-analysis of efficacy factors. **(A)** Network diagram of efficacy factors; **(B)** forest plot of efficacy factors.

Seven studies (Ruihong et al., 2021; Ding Guisheng, 2019; Xiaoli, 2018; Cao Zhengjun, 2017; Wu Xiangrong, 2016; Zhang et al., 2014; Zhang Xin, 2012) involving 1,018 patients examined efficacy factors, comparing six treatment modalities (Figure 4A). The following combinations demonstrated lower efficacy factors than HIFU alone: HIFU combined with GnRH-a (MD = −31%, 95% CI: −90 to −13), HIFU combined with ethanol (MD = −2.8%, 95% CI: −18 to 12), HIFU combined with a microbubble contrast agent (MD = −3.7%, 95% CI: −19 to 11), HIFU combined with oxytocin (MD = −7.8%, 95% CI: −15% to −1.2%), and HIFU combined with a microbubble contrast agent plus oxytocin (MD = −7.9%, 95% CI: −22% to 6.9%) (Figure 4B). Ranking probability analysis indicated that among the six treatment groups, HIFU combined with GnRH-a demonstrated the lowest efficacy factor (SUCRA = 89.8%, I^2^ = 11%; Table 3). Results of pairwise comparisons are presented in Supplementary Table S3.

### Secondary outcome

#### Sonication energy

**FIGURE 5:**
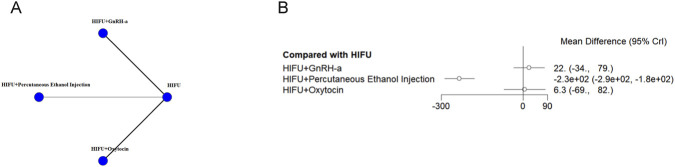
A network meta-analysis of sonication energy. **(A)** Network diagram of sonication energy; **(B)** forest plot of sonication energy.

Five studies (Wu Na, 2022; Ding Guisheng, 2019; Cao Zhengjun, 2017; Zhang et al., 2014; Zhang Xin, 2013) involving 427 patients examined sonication energy and compared four treatment modalities (Figure 5A). The irradiation energies for HIFU combined with GnRH-a (MD = 22J, 95% CI: −34–79), HIFU combined with ethanol (MD = −230J, 95% CI: −290 to −180), and HIFU combined with oxytocin (MD = 6.3J, 95% CI: −69–82) were all lower than that of HIFU alone (Figure 5B). Ranked probability analysis indicated that the lowest sonication energy among the four treatments was observed in the HIFU combined with ethanol group (SUCRA = 100%, I^2^ = 47%; Table 3). Results of pairwise comparisons are presented in Supplementary Table S4.

#### Irradiation duration

Ten studies (Chen Meiqin, 2023; Jia Xiao-Ping, 2023; Wu Na, 2022; Ruihong et al., 2021; Wang Yi, 2020; Ding Guisheng, 2019; Cao Zhengjun, 2017; Lingxian, 2016; Zhang et al., 2014; Zhang Xin, 2013) involving 1,183 patients examined irradiation duration across seven treatment modalities. HIFU combined with GnRH-a (MD = −270%, 95% CI: −560 to −8.2), testosterone propionate (MD = 47%, 95% CI: −470–570), ethanol (MD = −260%, 95% CI: −810 to 280), a microbubble contrast agent (MD = −270%, 95% CI: −570 to 28), oxytocin (MD = −130%, 95% CI: −400 to 180), and a microbubble contrast agent plus oxytocin (MD = −670%, 95% CI: −1,200 to −150) all demonstrated shorter irradiation times compared to HIFU alone. Ranking probability analysis indicated that the group receiving HIFU combined with a microbubble contrast agent plus oxytocin had the shortest irradiation time (SUCRA = 94.3%, I^2^ = 3%; Table 3). The network diagram and forest plot are shown in Supplementary Figures S1A,B. Results of pairwise comparisons are presented in Supplementary Table S5.

#### Treatment power

No statistically significant differences existed between interventions regarding treatment power (I^2^ = 5%; Table 3). The network diagram and forest plot are shown in Supplementary Figures S2A,B.

#### Treatment duration

Eleven studies (Song Xuewei, 2024; Chen Meiqin, 2023; Wu Na, 2022; Zhang Ting, 2021; Wang Yi, 2020; Ding Guisheng, 2019; Xiaoli, 2018; Lingxian, 2016; Wu Xiangrong, 2016; Zhang et al., 2014; Zhang Xin, 2013) involving 999 patients assessed treatment duration and compared six treatment modalities. HIFU combined with GnRH-a (MD = −20%, 95% CI: −37 to −2.4), testosterone propionate (MD = −2.5%, 95% CI: −36 to 31), PEI (MD = −44%, 95% CI: −80 to −7.8), a microbubble contrast agent (MD = −34%, 95% CI: −59 to −9.5), and oxytocin (MD = −10%, 95% CI: −26 to −7.5) all showed shorter treatment times than HIFU alone. Probability ranking analysis revealed that the HIFU combined with PEI group had the shortest treatment time (SUCRA = 90.1%, I^2^ = 3%; Table 3). The network diagram and forest plot are shown in Supplementary Figures S3A,B. Results of pairwise comparisons are presented in Supplementary Table S6.

#### Time to clearance per unit volume

Six studies (Xiaoli, 2018; Cao Zhengjun, 2017; Wu Xiangrong, 2016; Zhang et al., 2014; Zhang Xin, 2013; Zhang Xin, 2012) involving 657 patients investigated time to clearance per unit volume, comparing three treatment modalities. Treatment duration was shorter with HIFU combined with GnRH-a (MD = −2.3%, 95% CI: −5.9 to 1.6) and oxytocin (MD = −0.042, 95% CI: −0.049 to −0.035) compared to HIFU alone. Ranked probability analysis indicated that the HIFU plus oxytocin group achieved the shortest time to elimination per unit volume among the three treatments (SUCRA = 84.4%, I^2^ = 37%; Table 3). The network diagram and forest plot are shown in Supplementary Figures S4A,B. Results of pairwise comparisons are presented in Supplementary Table S7.

#### Presence of target area grey-scale changes

Three studies (Song Xuewei, 2024; Zhang Ting, 2021; Lingxian, 2016) involving 224 patients examined the presence of target area grey-scale changes and compared three treatment modalities. The probability of target area grey-scale changes was higher with HIFU combined with GnRH-a (RR = 1.1%, 95% CI: 0.93–1.2) and HIFU combined with testosterone propionate (MD = 1.4, 95% CI: 1.1–1.9) compared to HIFU alone. Ranked probability analysis indicated that the HIFU plus testosterone propionate group exhibited the highest probability of target area grey-scale change among the three treatments (SUCRA = 97.6%, I^2^ = 0%; Table 3). The network diagram and forest plot are shown in Supplementary Figures S5A,B. Results of pairwise comparisons are presented in Supplementary Table S8.

### Adverse events

In the 16 randomized controlled trials included in this study, 10 reported (Chen Meiqin, 2023; Jia Xiao-Ping, 2023; Wu Na, 2022; Li Fengli, 2021; Ruihong et al., 2021; Wang Yi, 2020; Ding Guisheng, 2019; Zhang et al., 2014; Zhang Xin, 2013; Zhang Xin, 2012) safety data, which were incorporated into the adverse reaction analysis. Meta-analyses were conducted for sacral pain, treatment site pain, radiating pain, leg pain, lower abdominal pain, and fever. HIFU combined with a microbubble contrast agent showed a lower incidence of treatment site pain compared to other therapies (RR = 0.66%, 95% CI: 0.45–0.90, SUCRA = 99.5%, I^2^ = 0%). The network diagram and forest plot are shown in Supplementary Figures S6A,B, respectively. HIFU combined with oxytocin demonstrated a lower incidence of radiating pain compared to other therapies (RR = 0.44%, 95% CI: 0.18–0.94, SUCRA = 87.7%, I^2^ = 0%). No significant differences were observed between interventions for sacral pain (I^2^ = 0%), leg pain (I^2^ = 9%), lower abdominal pain (I^2^ = 14%), or fever (I^2^ = 0%) (Table 4). The network diagram and forest plot are shown in Supplementary Figures S7A,B. The results of pairwise comparisons of pain at treatment site and radiating pain are shown in Supplementary Tables S10, S9.

**TABLE 4:** I^2^ and SUCRA values of adverse reactions.

Adverse reactions	I^2^ (%)	SUCRA (%)						
		HIFU	HIFU + GnRH-a	HIFU + testosterone propionate	HIFU + percutaneous ethanol injection	HIFU + microbubble contrast agent	HIFU + oxytocin	HIFU + microbubble contrast agent + oxytocin
Sacral pain	0	51.92	68.00	-	28.36	72.70	51.60	27.42
Pain at treatment site	0	53.62	39.76	-	-	99.48	7.15	-
Radiating pain	0	11.14	51.20	-	-	-	87.66	-
Leg pain	9	49.16	-	-	-	47.19	81.73	21.91
Lower abdominal pain	14	77.53	-	-	11.80	55.85	71.06	33.75
Fever	0	45.51	-	-	33.58	63.88	53.48	53.55

### Publication bias

For outcomes including sonication energy, grayscale changes in target area, pain at treatment site, radiating pain, and time to elimination per unit volume the number of included studies was too small to perform Egger’s test for publication bias; however, qualitative inspection of funnel plots suggested no evidence of publication bias. Similarly, qualitative assessment of funnel plots for NPVR (p = 0.19), EEF (p = 0.55), sonication time (p = 0.26), and treatment time (p = 0.13) also indicated no evidence of publication bias. The funnel plots for publication bias are presented in the Supplementary Figures S8–S16.

## Discussion

This study primarily conducted a network meta-analysis on HIFU combined with various drug therapies. Analysis of clinical data from 16 randomized controlled trials (RCTs) involving 1,685 patients revealed the following findings: HIFU combined with ethanol ablation demonstrated higher NPVR, lower sonication energy, and shorter treatment durations; HIFU combined with GnRH-a exhibited a lower energy efficiency factor. Because there were no closed loops in this study, inconsistency tests could not be performed. It should be noted that the units of outcome measures across studies were generally consistent; for the few cases where units differed (e.g., Treatment Power, Sonication time for ablating 1 mm^3^ of lesion), we performed appropriate conversions to unify them to J and s, respectively. Moreover, the baseline levels of the included studies were similar, therefore the mean difference (MD) can directly reflect the intervention effect. Because Egger’s test could not be performed for some dichotomous outcome indicators due to an insufficient number of studies, only qualitative visual inspection was possible, which indicated no publication bias. None of the continuous variables showed evidence of publication bias.

Adenomyosis is a common benign gynecological condition that significantly impacts patients’ daily lives, yet its pathogenesis remains unclear. Even after surgical intervention, the prevalence of adenomyosis among consecutive hysterectomy patients over the past 50 years has ranged from 8.8% to 61.5% (Upson and Missmer, 2020), resulting in considerable distress. As a non-invasive technique, HIFU is increasingly applied clinically with notable efficacy (O'Reilly, 2024). HIFU operates by using an external ultrasound generator to precisely focus high-frequency ultrasound waves onto target tissue within the body. Through mechanisms such as thermal and cavitation effects, it generates temperatures exceeding 55 °C at the focal point, causing instantaneous coagulative necrosis and effective non-invasive tissue ablation (Li et al., 2006). The combination of HIFU with drug therapy for adenomyosis has also been found to offer additional advantages, with its mechanisms and precise efficacy warranting further attention.

Li et al. (Jeng et al., 2020) observed that HIFU combined with PEI significantly reduced both uterine volume and lesion size, achieving an overall efficacy rate of 94.23%. Research indicates injecting PEI prior to radiofrequency ablation directly damages vascular endothelium, triggering platelet aggregation and subsequent microthrombus formation to embolize vessels. Concurrently, these microthrombi occlude the feeding vessels of the lesion, diminishing the “heat sink effect,” whereby blood flow dissipates heat. This allows the heat generated during the procedure to concentrate more intensely and efficiently on the target lesion, resulting in larger and more complete ablation volumes. This process interrupts blood flow around the lesion, enhances thermal ablation efficacy, reduces the energy required for irradiation, and shortens treatment duration—findings consistent with the present study. Additional studies indicate that high concentrations of PEI can improve thermal ablation heat dissipation by increasing tissue necrosis. Furthermore, PEI offers economic advantages due to its low intrinsic cost and reduced wear on HIFU equipment (León-Salas et al., 2023), making it more conducive to widespread adoption. Microbubble contrast agents enable exceptionally precise delineation of target ablation zones during HIFU therapy, effectively avoiding damage to surrounding healthy tissue. This precision is particularly crucial for lesions with rich blood supplies or ill-defined borders. Moreover, the cavitation effect of microbubbles significantly reduces the energy and time required for ablation while enhancing thermal efficacy (Klibanov, 2006; Chen et al., 2022).

In the study by Yao et al., separate observations were made on HIFU alone, HIFU combined with oxytocin, HIFU combined with the microbubble contrast agent SonoVue, and HIFU combined with both SonoVue and oxytocin. The findings revealed a significantly reduced ablation time and energy efficiency factor (EEF) in the group receiving HIFU combined with the microbubble contrast agent and oxytocin (*p* < 0.05). Microbubble contrast agents are now well-established in HIFU treatment for uterine fibroids and adenomyosis. With the introduction of microbubbles, effective therapeutic temperatures are achieved with lower ultrasound energy or shorter irradiation times (Isern et al., 2015; Cheng et al., 2017). Oxytocin constricts uterine arteries, reducing blood flow to adenomyotic lesions (Richter et al., 2004). Their combined use further shortens HIFU irradiation duration. The combination of HIFU and oxytocin decreases treatment duration, thereby minimizing unnecessary thermal stimulation of pain-sensing nerves in the treatment area and surrounding normal tissues. This aligns with our findings, which indicate a reduced incidence of treatment-site pain and neuropathic pain in this context. Additionally, the microbubble contrast agent SonoVue is characterized by low solubility in blood and high stability (Chen et al., 2022), providing a distinct advantage over other microbubble contrast agents.

GnRH-a, commonly used as a hormonal agent for treating adenomyosis, suppresses the pituitary-gonadal axis to reduce estrogen levels, leading to atrophy and volume reduction in estrogen-dependent uterine fibroids and adenomyotic lesions (Tesone et al., 2008). The energy efficiency factor is considered one of the “gold standards” for assessing HIFU efficacy (Chen et al., 2022). When acoustic power and irradiation time are kept constant, lesion reduction under the influence of GnRH-a decreases the energy efficiency factor. This finding observation is consistent with a previous meta-analysis by Li et al., which demonstrated a higher rate of uterine volume reduction in the HIFU plus GnRH-a group compared to the HIFU-alone group.

Our findings suggest that combining HIFU with various pharmacological treatments for adenomyosis offers distinct advantages and holds significant clinical value for future management. However, this network meta-analysis has several limitations. First, all included studies originate from China, which may introduce regional heterogeneity. Second, the lack of standardization among practitioners across different clinical trials has resulted in variations in the treatment techniques administered to individual patients. Third, the involvement of three distinct HIFU device manufacturers in this network meta-analysis may have introduced differences in treatment efficacy. Additionally, the included studies were limited to small-scale clinical trials (16 studies involving 1,685 patients), which may present the limitation of a small sample size. Future randomized controlled trials should aim to increase sample sizes, involve large sample populations, rigorously document adverse events, and strictly adhere to blinding protocols to minimize errors and bias.

## Conclusion

Based on the results of a comprehensive analysis, HIFU combined with ethanol ablation achieved higher NPVR, lower sonication energy, and shorter treatment duration. HIFU combined with GnRH-a demonstrated lower energy efficiency factors. It is imperative to consider multiple factors when selecting specific medications, including robust evidence from high-quality research, the patient’s condition and status, and the clinical experience of healthcare practitioners.

## References

[B1] Bahutair S. N. Alhubaishi L. Y. (2024). High-intensity focused ultrasound in adenomyosis treatment: insights on safety, efficacy, and reproductive prospects. Womens Health 20, 17455057241295593. 10.1177/17455057241295593 39494764 PMC11536486

[B2] Cao Zhengjun A. X. L. (2017). Intensity focused ultrasound combined with GnRH a for treatment ofuterine adenomyosis: A dosimetric study. J. Tongji Univ. Med. Sci. 38 (02), 56–59. 10.16118/j.1008-0392.2017.02.011

[B3] Capezzuoli T. Toscano F. Ceccaroni M. Roviglione G. Stepniewska A. Fambrini M. (2024). Conservative surgical treatment for adenomyosis: new options for looking beyond uterus removal. Best. Pract. Res. Clin. Obstet. Gynaecol. 95, 102507. 10.1016/j.bpobgyn.2024.102507 38906739

[B4] Carter C. S. Kenkel W. M. Maclean E. L. Wilson S. R. Perkeybile A. M. Yee J. R. (2020). Is oxytocin Nature's Mmdicine. Pharmacol. Rev. 72 (4), 829–861. 10.1124/pr.120.019398 32912963 PMC7495339

[B5] Chen C. Liu Y. Maruvada S. Myers M. Khismatullin D. (2012). Effect of ethanol injection on cavitation and heating of tissues exposed to high-intensity focused ultrasound. Phys. Med. Biol. 57 (4), 937–961. 10.1088/0031-9155/57/4/937 22290554

[B6] Chen L. Dong B. Jiang L. Zhang J. Chen L. Li T. (2022). Microbubble contrast agent SonoVue: an efficient medium for the preoperative lymphatic mapping of thyroid carcinoma. Front. Bioeng. Biotechnol. 10, 1077145. 10.3389/fbioe.2022.1077145 36568294 PMC9773067

[B7] Chen W. C. Chang T. C. Perera L. Cheng M. H. Hong J. J. Cheng C. M. (2024). Pilot study on the impact of HIFU treatment on miRNA profiles in vaginal secretions of uterine fibroids and adenomyosis patients. Int. J. Hyperth. 41 (1), 2418426. 10.1080/02656736.2024.2418426 39462514

[B8] Chen Meiqin S. C. G. P. (2023). Effectiveness and safety analysis of microbubbleultrasound contrast agent assisted high intensity focusedultrasound in the treatment of adenomyosis. Contemp. Med. 29 (31), 68–72. 10.3969/j.issn.1009-4393.2023.31.018

[B9] Cheng C. Xiao Z. Huang G. Zhang L. Bai J. (2017). Enhancing ablation effects of a microbubble contrast agent on high-intensity focused ultrasound: an experimental and clinical study. BJOG 124 (Suppl. 3), 78–86. 10.1111/1471-0528.14744 28856858

[B10] Ding Guisheng C. S. L. J. (2019). Clinical value of high intensity focused ultrasound combined with percutaneous ethanol injection in treatment of adenomyosis. J. Clin. Ultrasound Med. 21 (05), 385–387. 10.16245/j.cnki.issn1008-6978.2019.05.027

[B11] Dong X. Yang Z. (2010). High-intensity focused ultrasound ablation of uterine localized adenomyosis. Curr. Opin. Obstet. Gynecol. 22 (4), 326–330. 10.1097/GCO.0b013e32833bea2e 20610999

[B12] Dueholm M. (2018). Minimally invasive treatment of adenomyosis. Best. Pract. Res. Clin. Obstet. Gynaecol. 51, 119–137. 10.1016/j.bpobgyn.2018.01.016 29555380

[B13] Grow D. R. Filer R. B. (1991). Treatment of adenomyosis with long-term GnRH analogues: a case report. Obstet. Gynecol. 78 (3 Pt 2), 538–539. 1908069

[B14] Harada T. Khine Y. M. Kaponis A. Nikellis T. Decavalas G. Taniguchi F. (2016). The impact of adenomyosis on women's fertility. Obstet. Gynecol. Surv. 71 (9), 557–568. 10.1097/OGX.0000000000000346 27640610 PMC5049976

[B15] Hermesch A. C. Kernberg A. S. Layoun V. R. Caughey A. B. (2024). Oxytocin: physiology, pharmacology, and clinical application for labor management. Am. J. Obstet. Gynecol. 230 (3S), S729–S739. 10.1016/j.ajog.2023.06.041 37460365

[B16] Isern J. Pessarrodona A. Rodriguez J. Vallejo E. Gimenez N. Cassadó J. (2015). Using microbubble sonographic contrast agent to enhance the effect of high intensity focused ultrasound for the treatment of uterine fibroids. Ultrason. Sonochem. 27, 688–693. 10.1016/j.ultsonch.2015.05.027 26113390

[B17] Jeng C. J. Ou K. Y. Long C. Y. Chuang L. Ker C. R. (2020). 500 cases of high-intensity focused ultrasound (HIFU) ablated uterine fibroids and adenomyosis. Taiwan. J. Obstet. Gynecol. 59 (6), 865–871. 10.1016/j.tjog.2020.09.013 33218403

[B18] Jia Xiao-Ping H. J. C. Y. (2023). Evaluation of the effect of pretreatment with GnRH-a before HIUtreatment for severe adenomyosis. Chin. J. Fam. Plan. Obstetrics and Gynecol. 15 (05), 90–94. 10.3969/j.issn.1674-4020.2023.05.19

[B44] Klibanov A. L. (2006). Microbubble contrast agents: targeted ultrasound imaging and ultrasound-assisted drug-delivery applications. Invest Radiol. 41 (3), 354–362. 10.1097/01.rli.0000199292.88189.0f 16481920

[B20] León-Salas B. Hernández-Yumar A. Infante-Ventura D. de Armas C. A. González H. Y. Linertová R. (2023). Percutaneous ethanol injection in thyroid nodular pathology and metastatic cervical adenopathies: a systematic review, meta-analysis and economic evaluation. Endocrinol. Diabetes Nutr. 70 (9), 572–583. 10.1016/j.endien.2023.08.007 37996202

[B21] Li F. Wang Z. Du Y. Ma P. Bai J. Wu F. (2006). Study on therapeutic dosimetry of HIFU ablation tissue. Sheng Wu Yi Xue Gong Cheng Xue Za Zhi 23 (4), 839–843. 17002121

[B22] Li Fengli Y. X. W. Y. (2021). Effects of absolute ethanol injection combined with high intensity focused ultrasound in treatment of patients with adenomyosis. China J. Health Psychol. 33 (22), 57–58+61. 10.3969/j.issn.1672-0369.2021.22.021

[B23] Lingxian W. (2016). Observation on the application value of testosterone propionate in high-intensity focused ultrasound treatment for adenomyosis. Maternal Child World 2016 (2), 12.

[B24] Naftalin J. Hoo W. Pateman K. Mavrelos D. Holland T. Jurkovic D. (2012). How common is adenomyosis? A prospective study of prevalence using transvaginal ultrasound in a gynaecology clinic. Hum. Reprod. 27 (12), 3432–3439. 10.1093/humrep/des332 23001775

[B25] O'Reilly M. A. (2024). Exploiting the mechanical effects of ultrasound for noninvasive therapy. Science 385 (6714), eadp7206. 10.1126/science.adp7206 39265013

[B26] Pang L. L. Mei J. Fan L. X. Zhao T. T. Li R. N. Wen Y. (2021). Efficacy of high-intensity focused ultrasound combined with GnRH-a for adenomyosis: a systematic review and meta-analysis. Front. Public Health 9, 688264. 10.3389/fpubh.2021.688264 34485218 PMC8415267

[B27] Peng Y. Dai Y. Yu G. Yang X. Wen C. Jin P. (2021). Clinical evaluation of HIFU combined with GnRH-a and LNG-IUS for adenomyosis patients who failed to respond to drug therapies: two-year follow-up results. Int. Journal Hyperthermia 38 (1), 1271–1275. 10.1080/02656736.2021.1967467 34423729

[B28] Richter O. N. Kübler K. Schmolling J. Kupka M. Reinsberg J. Ulrich U. (2004). Oxytocin receptor gene expression of estrogen-stimulated human myometrium in extracorporeally perfused non-pregnant uteri. Mol. Hum. Reprod. 10 (5), 339–346. 10.1093/molehr/gah039 15044599

[B29] Ruihong Y. Wei Z. Bulang G. Jihong H. Tao W. (2021). Microbubble contrast agent SonoVue combined with oxytocin improves the efficiency of high-intensity focused ultrasound ablation for adenomyosis. Int. Journal Hyperthermia 38 (1), 1601–1608. 10.1080/02656736.2021.1993357 34763594

[B30] Song Xuewei Z. M. J. F. (2024). Clinical efficacy of GnRH-a pretreatment combined with HIFU ablation in the treatment of diffuse adenomyosis. Chin. J. Sexology 33 (09), 75–79. 10.3969/j.issn.1672-1993.2024.09.018

[B31] Tesone M. Bilotas M. Barañao R. I. Meresman G. (2008). The role of GnRH analogues in endometriosis-associated apoptosis and angiogenesis. Gynecol. Obstet. Invest. 66 (Suppl. 1), 10–18. 10.1159/000148026 18936547

[B32] Upson K. Missmer S. A. (2020). Epidemiology of adenomyosis. Semin. Reprod. Med. 38 (2-03), 89–107. 10.1055/s-0040-1718920 33105509 PMC7927213

[B33] Wang Yi C. J. H. H. (2020). Clinical value of sulfur hexafluoride microbubbles in HIFU treatment of uterine adenomyosis. New Med. 51 (07), 548–552. 10.3969/j.issn.0253-9802.2020.07.012

[B34] Wu Na Y. N. D. J. (2022). Clinical efficacy and safety of leuprorelin combined with high intensity focused ultrasound for treating adenomyosis. Chin. J. Fam. Plan. 30 (03), 547–550+555. 10.3969/j.issn.1004-8189.2022.03.012

[B35] Wu Xiangrong Z. Y. Z. H. (2016). Application of oxytocin in ultrasound ablation treatment for 48 cases of adenomyosis. Chin. Ethn. Med. Folk Med. 25 (04), 100+102.

[B36] Xiaoli G. (2018). Analysis of the efficacy and advantages of oxytocin application in ultrasound ablation therapy for adenomyosis. Heilongjiang Med. J. 31 (04), 813–814. 10.14035/j.cnki.hljyy.2018.04.047

[B37] Younes G. Tulandi T. (2018). Conservative surgery for adenomyosis and results: A systematic review. J. Minim. Invasive Gynecol. 25 (2), 265–276. 10.1016/j.jmig.2017.07.014 28739414

[B38] Yu O. Schulze-Rath R. Grafton J. Hansen K. Scholes D. Reed S. D. (2020). Adenomyosis incidence, prevalence and treatment: united States population-based study 2006-2015. Am. J. Obstet. Gynecol. 223 (1), 94.e1–94.e10. 10.1016/j.ajog.2020.01.016 31954156

[B39] Zhang X. Zou M. Zhang C. He J. Mao S. Wu Q. (2014). Effects of oxytocin on high intensity focused ultrasound (HIFU) ablation of adenomysis: A prospective study. Eur. J. Radiol. 83 (9), 1607–1611. 10.1016/j.ejrad.2014.05.008 24951357

[B40] Zhang R. Zhang Y. Yang Z. Chen J. Wang Q. Ma J. (2022). The safety and efficacy of add-on use of oxytocin in uterine leiomyoma patients undergoing high-intensity focused ultrasound and ultrasound-guided intratumoral ethanol injection: A randomized controlled trial. Ann. Palliat. Med. 11 (6), 2033–2042. 10.21037/apm-22-602 35817738

[B41] Zhang Ting Q. Y. P. L. (2021). Clinical efficacy of high-intensity focused ultrasound ablation combined with GnRH-a in the treatment of diffuse adenomyosis. J. Guangxi Med. Univ. 38 (12), 2347–2351. 10.16190/j.cnki.45-1211/r.2021.12.024

[B42] Zhang Xin H. J. M. S. (2012). Effeets of oxytocin on high intensity focused ultrasound ablation of adenomysis. Chongqing Med. 41 (36), 3825–3827. 10.3969/j.issn.1671-8348.2012.36.010

[B43] Zhang Xin H. J. M. S. (2013). Dose-effect research on the effect of oxytocin on high intensity focused ultrasound ablation of adenomysis. J. Lasers. 34 (06), 103–104.

