# Supplementary material for: The efficacy of ultrasonic ablation combined with medication in the treatment of adenomyosis: a systematic review and network meta-analysis
Source: Front Pharmacol. 2026 Jun 12;17:1767202. doi: 10.3389/fphar.2026.1767202 (PMC13299098; doi:10.3389/fphar.2026.1767202)
Supplement: Supplementary file 1 [file Supplementaryfile1.docx]

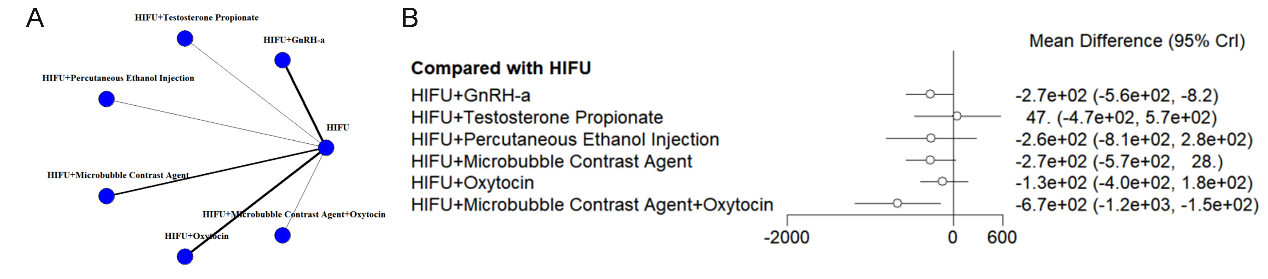


Figure S1 (A) Network diagram of irradiation duration, (B) Forest plot of irradiation duration


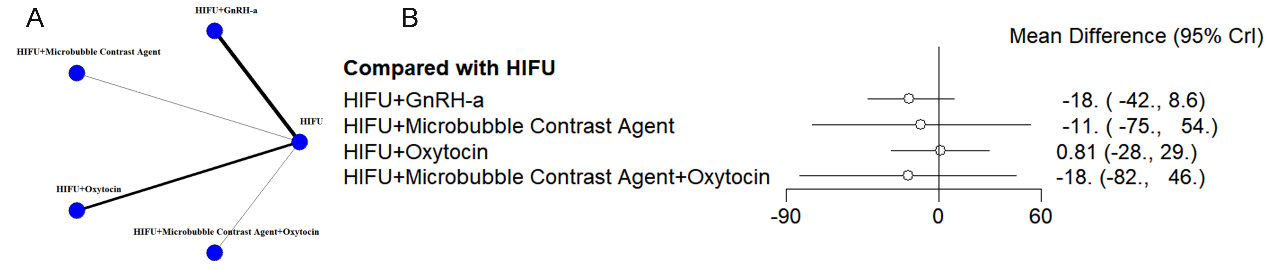


Figure S2 (A) Network diagram of treatment power, (B) Forest plot of treatment power


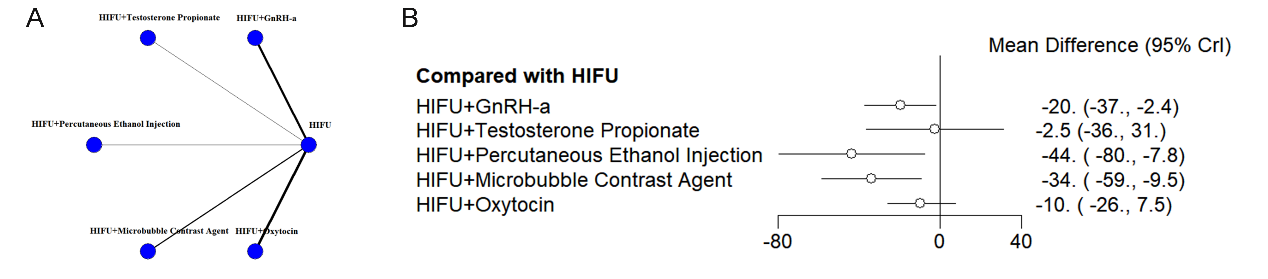


Figure S3 (A) Network diagram of treatment time, (B) Forest plot of treatment time


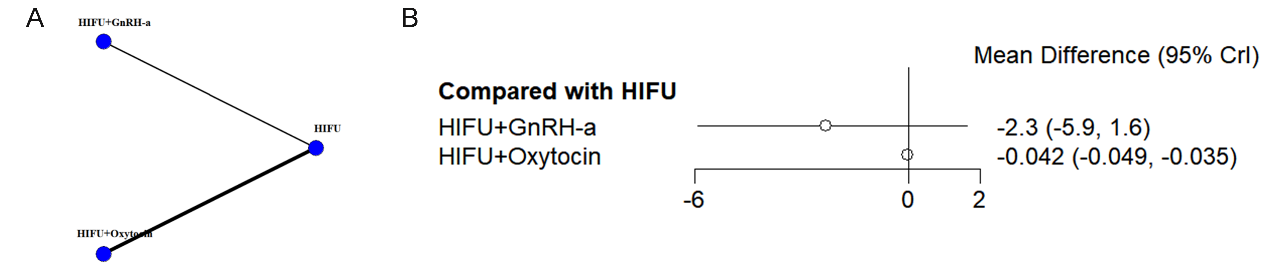


Figure S4 (A) Network diagram of time to elimination per unit volume, (B) Forest plot of time to elimination per unit volume


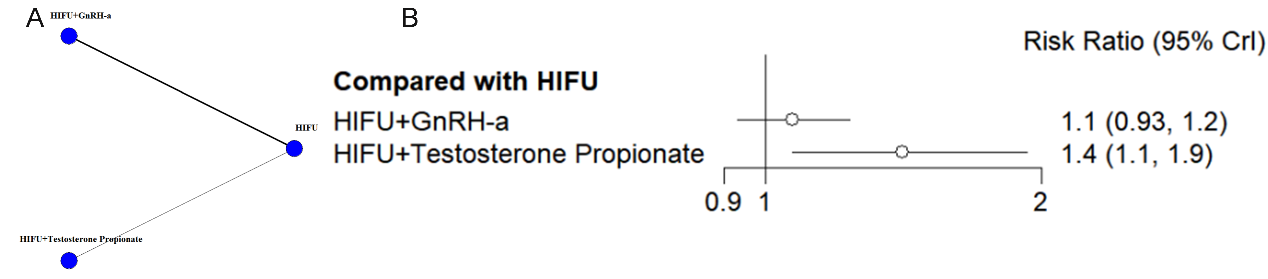


Figure S5 (A) Network diagram of target area grey-scale changes, (B) Forest plot of target area grey-scale changes


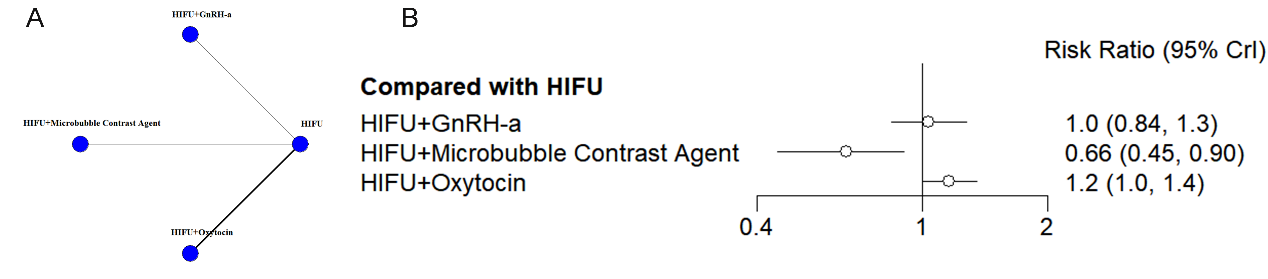


Figure S6 (A) Network diagram of pain at treatment site, (B) Forest plot of pain at treatment site


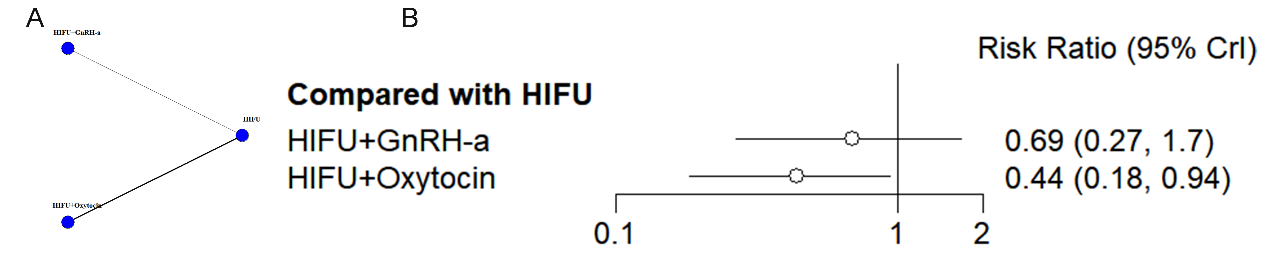


Figure S7 (A) Network diagram of radiating pain, (B) Forest plot of radiating pain


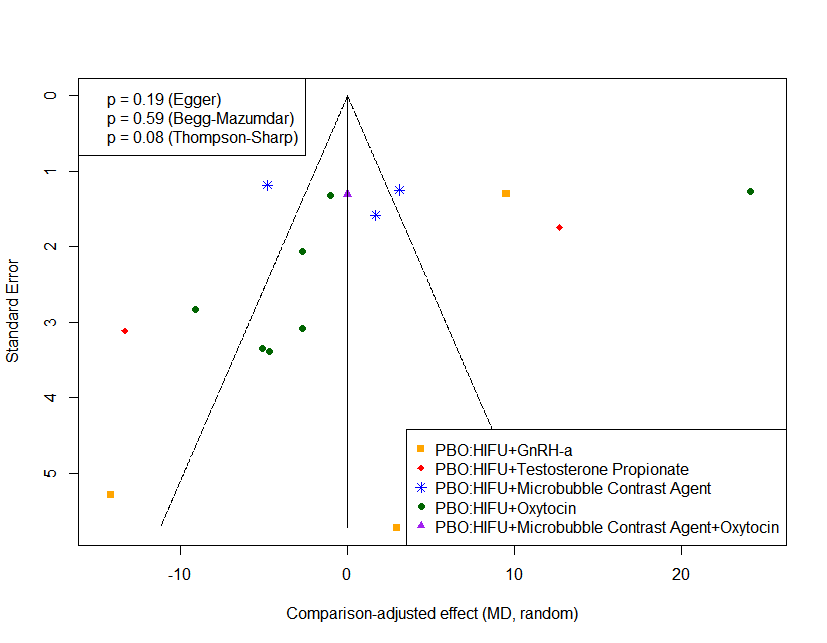


Figure S8 The funnel plot for NPVR


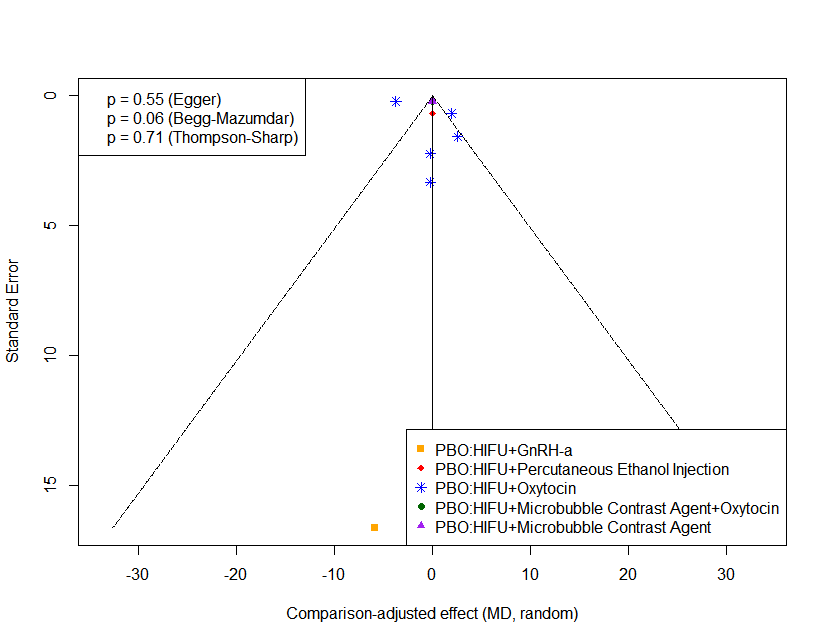


Figure S9 The funnel plot for EEF


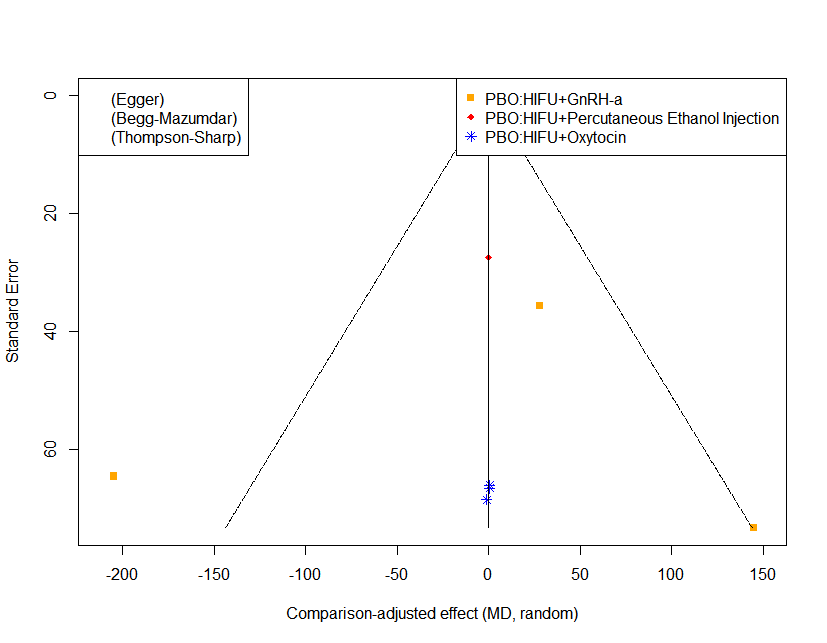


Figure S10 The funnel plot for Sonication energy


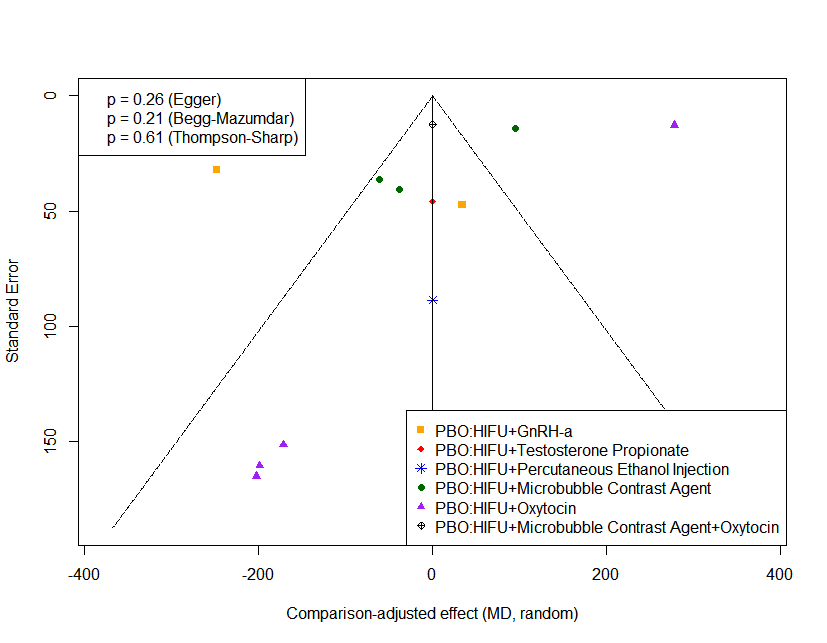


Figure S11 The funnel plot for Sonication time


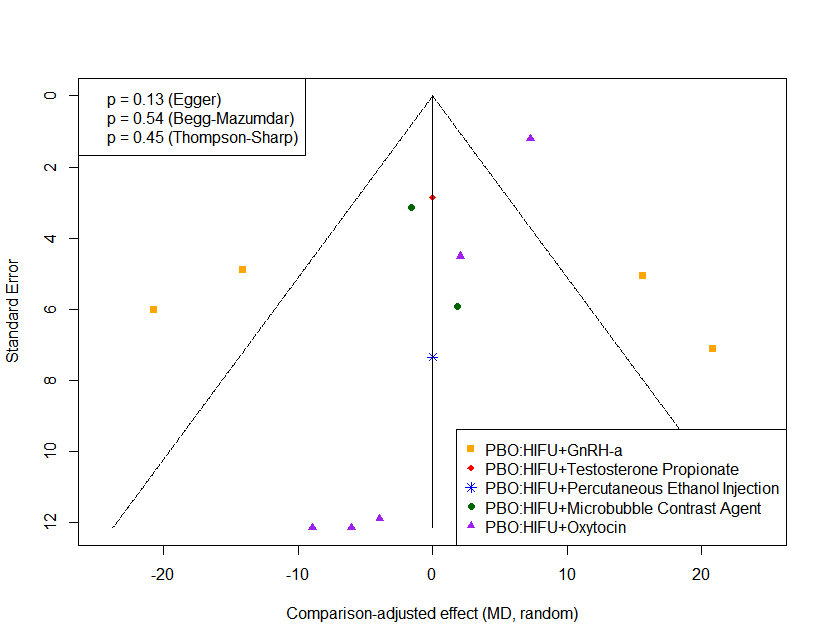


Figure S12 The funnel plot for Treatment time


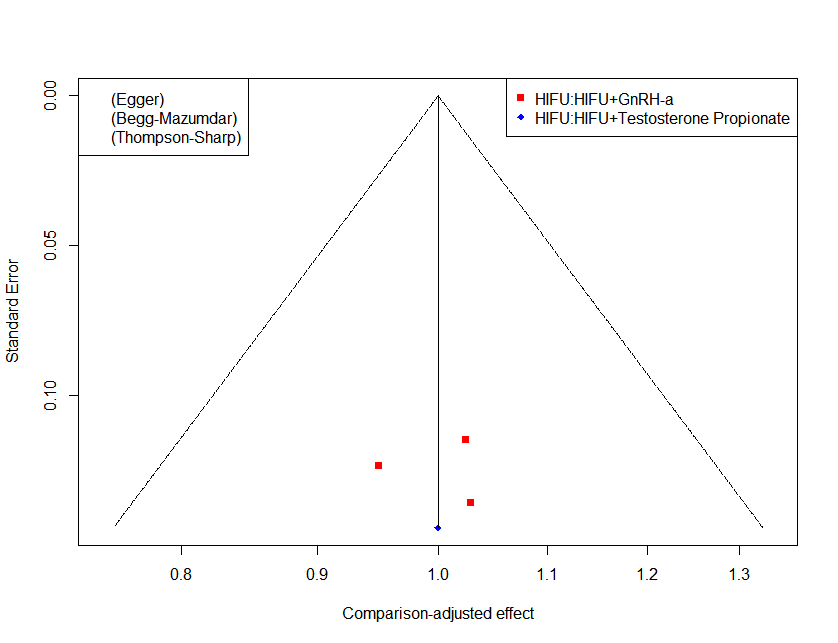


Figure S13 The funnel plot for Grayscale changes in target area


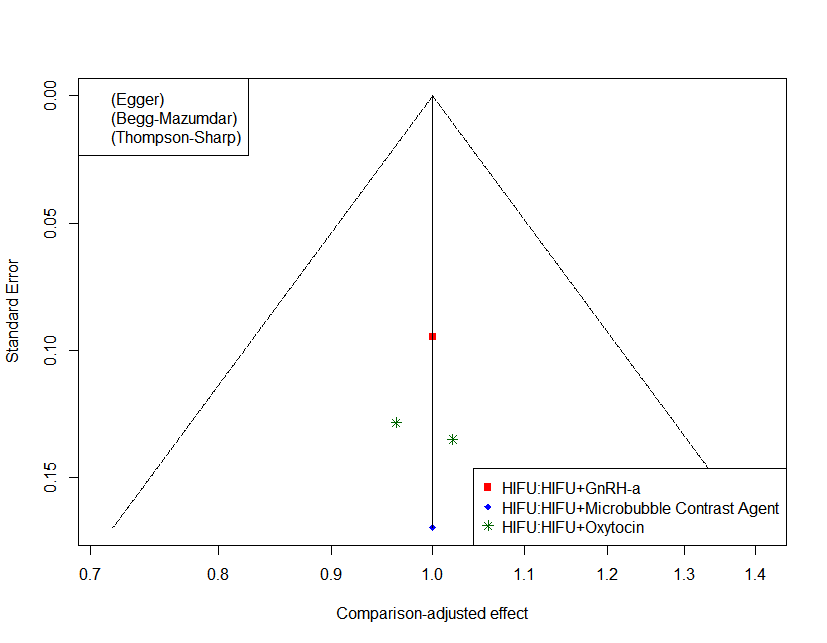


Figure S14 The funnel plot for Pain at treatment site


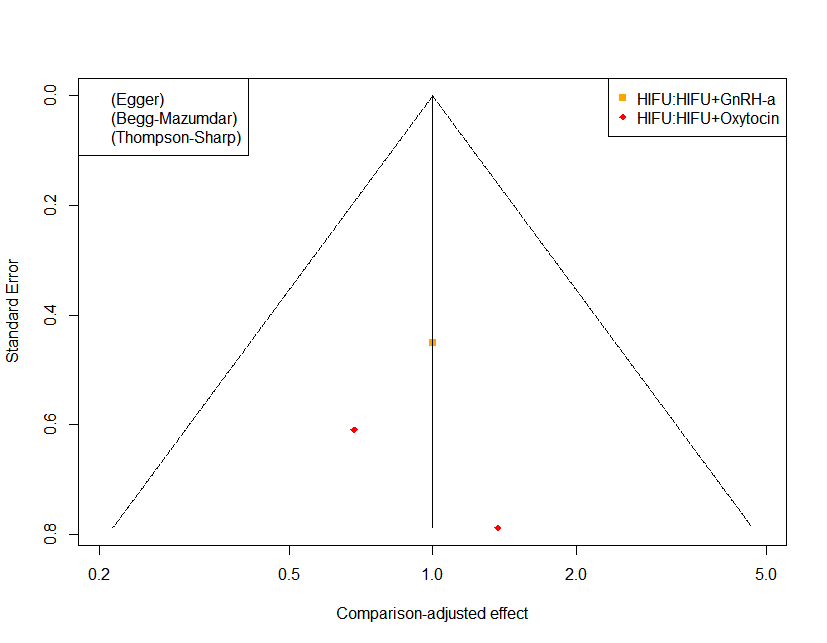


Figure S15 The funnel plot for Radiating pain


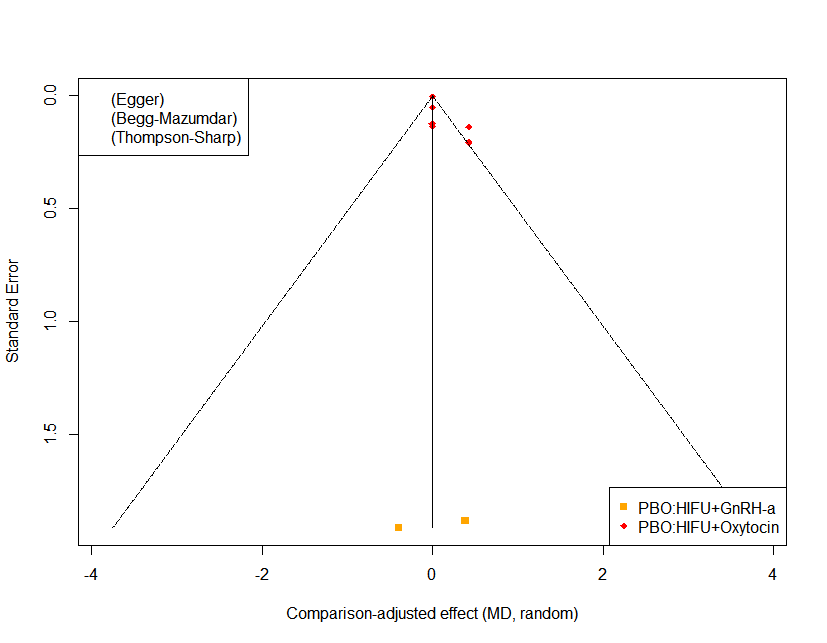


Figure S16 The funnel plot for time to elimination per unit volume

Table S1 Search method

| Databases | Search strategy | Results | Date |
| --- | --- | --- | --- |
| Web of science | ((“High Intensity Focused Ultrasound Ablation" OR "HIFU" OR "high intense focused ultrasound" OR "high intensity focused ultrasonic ablation" OR "high intensity focused ultrasonic treatment" OR "high intensity focused ultrasound" OR "high intensity focused ultrasound ablation")) AND (( “adenomyosis uteri" OR "endometrial adenoma" OR "endometriosis interna" OR "endometriosis uterina" OR "internal endometriosis" OR "uterine adenomyosis" OR "uterus adenomyosis" OR "adenomyosis")) AND ("adenomyosis uteri" OR "endometrial adenoma" OR "endometriosis interna" OR "endometriosis uterina" OR "internal endometriosis" OR "uterine adenomyosis" OR "uterus adenomyosis" OR "adenomyosis")) | 221 | 2025.06.23 |
| PubMed | (("adenomyosis"[MeSH Terms]) OR ("adenomyosis uteri"[Title/Abstract] OR "endometrial adenoma"[Title/Abstract] OR "endometriosis interna"[Title/Abstract] OR "internal endometriosis"[Title/Abstract] OR "uterine adenomyosis"[Title/Abstract] OR "uterus adenomyosis"[Title/Abstract] OR "adenomyosis"[Title/Abstract])) AND (("high intensity focused ultrasound ablation"[MeSH Terms]) OR ("High Intensity Focused Ultrasound Ablation"[Title/Abstract] OR "HIFU"[Title/Abstract] OR "high intense focused ultrasound"[Title/Abstract] OR "high intensity focused ultrasound"[Title/Abstract] OR "High Intensity Focused Ultrasound Ablation"[Title/Abstract])) | 156 | 2025.06.23 |
| cochrane | ((MeSH descriptor: [Adenomyosis] explode all trees) OR ((‘adenomyosis uteri' OR 'endometrial adenoma' OR 'endometriosis interna' OR 'endometriosis uterina' OR 'internal endometriosis' OR 'uterine adenomyosis' OR 'uterus adenomyosis' OR 'adenomyosis’):ti,ab,kw)) AND ((MeSH descriptor: [High-Intensity Focused Ultrasound Ablation] explode all trees) OR ((‘High Intensity Focused Ultrasound Ablation' OR 'HIFU' OR 'high intense focused ultrasound' OR 'high intensity focused ultrasonic ablation' OR 'high intensity focused ultrasonic treatment' OR 'high intensity focused ultrasound' OR 'high intensity focused ultrasound ablation’):ti,ab,kw)) | 14 | 2025.06.23 |
| Embase | (('adenomyosis'/exp) OR ('adenomyosis uteri':ti,ab,kw OR 'endometrial adenoma':ti,ab,kw OR 'endometriosis interna':ti,ab,kw OR 'endometriosis uterina':ti,ab,kw OR 'internal endometriosis':ti,ab,kw OR 'uterine adenomyosis':ti,ab,kw OR 'uterus adenomyosis':ti,ab,kw OR 'adenomyosis':ti,ab,kw)) AND (('high intensity focused ultrasound'/exp) OR ('high intensity focused ultrasound ablation':ti,ab,kw OR 'hifu':ti,ab,kw OR 'high intense focused ultrasound':ti,ab,kw OR 'high intensity focused ultrasonic ablation':ti,ab,kw OR 'high intensity focused ultrasonic treatment':ti,ab,kw OR 'high intensity focused ultrasound':ti,ab,kw OR 'high intensity focused ultrasound ablation':ti,ab,kw)) | 262 | 2025.06.23 |
| SINOMED | ("超声"[常用字段:智能])AND(("子宫腺肌病"[常用字段:智能] OR"子宫肌腺症"[常用字段:智能] OR"内在性子宫内膜异位症"[常用字段:智能])) | 1436 | 2025.06.23 |
| CNKI | 主题:(‘子宫腺肌病’) AND 主题:(‘超声’) | 1559 | 2025.06.23 |
| WanFang | 主题:(“子宫腺肌病”) AND 主题:(“超声”) | 3003 | 2025.06.23 |
| VIP | 主题:(子宫腺肌病) AND 主题:(超声) | 1999 | 2025.06.23 |

Table S2 League table of NPVR

|  | HIFU | HIFU+GnRH-a | HIFU+Testosterone Propionate | HIFU+Percutaneous Ethanol Injection | HIFU+Microbubble Contrast Agent | HIFU+Oxytocin | HIFU+Microbubble Contrast Agent+Oxytocin |
| --- | --- | --- | --- | --- | --- | --- | --- |
| HIFU | - | 10.44 (-4.08, 25.45) | 1.45 (-22.8, 25.93) | **27.45 (2.66, 52.34)** | 4.07 (-9.94, 18.21) | 7.12 (-2.03, 16.52) | 9.96 (-14.3, 34.08) |
| HIFU+GnRH-a | -10.44 (-25.45, 4.08) | - | -8.94 (-37.69, 19.15) | 17.12 (-12.11, 45.64) | -6.31 (-26.83, 13.91) | -3.3 (-20.86, 14.13) | -0.44 (-29.07, 27.54) |
| HIFU+Testosterone Propionate | -1.45 (-25.93, 22.8) | 8.94 (-19.15, 37.69) | - | 26.03 (-8.61, 60.7) | 2.67 (-25.47, 30.59) | 5.69 (-20.44, 31.65) | 8.4 (-25.62, 42.83) |
| HIFU+Percutaneous Ethanol Injection | -27.45 (-52.34, -2.66) | -17.12 (-45.64, 12.11) | -26.03 (-60.7, 8.61) | - | -23.43 (-51.83, 5.02) | -20.36 (-46.62, 6.34) | -17.51 (-52.44, 17.23) |
| HIFU+Microbubble Contrast Agent | -4.07 (-18.21, 9.94) | 6.31 (-13.91, 26.83) | -2.67 (-30.59, 25.47) | 23.43 (-5.02, 51.83) | - | 3.05 (-13.67, 19.99) | 5.87 (-22.28, 33.62) |
| HIFU+Oxytocin | -7.12 (-16.52, 2.03) | 3.3 (-14.13, 20.86) | -5.69 (-31.65, 20.44) | 20.36 (-6.34, 46.62) | -3.05 (-19.99, 13.67) | - | 2.81 (-23.34, 28.63) |
| HIFU+Microbubble Contrast Agent+Oxytocin | -9.96 (-34.08, 14.3) | 0.44 (-27.54, 29.07) | -8.4 (-42.83, 25.62) | 17.51 (-17.23, 52.44) | -5.87 (-33.62, 22.28) | -2.81 (-28.63, 23.34) | - |

Table S3 League table of EEF

|  | HIFU | HIFU+GnRH-a | HIFU+Percutaneous Ethanol Injection | HIFU+Microbubble Contrast Agent | HIFU+Oxytocin | HIFU+Microbubble Contrast Agent+Oxytocin |
| --- | --- | --- | --- | --- | --- | --- |
| HIFU | - | **-30.75 (-89.52, -13.07)** | -2.79 (-17.81, 11.91) | -3.73 (-18.8, 10.92) | -7.77 (-14.68, -1.2) | -7.89 (-22.32, 6.92) |
| HIFU+GnRH-a | 30.75 (13.07, 89.52) | - | 28.21 (7.71, 90.72) | 27.28 (6.7, 89.81) | 23.05 (4.63, 82.72) | 23.1 (2.62, 85.93) |
| HIFU+Percutaneous Ethanol Injection | 2.79 (-11.91, 17.81) | -28.21 (-90.72, -7.71) | - | -0.91 (-22.04, 20.15) | -4.99 (-21.07, 11.19) | -5.07 (-25.72, 16.07) |
| HIFU+Microbubble Contrast Agent | 3.73 (-10.92, 18.8) | -27.28 (-89.81, -6.7) | 0.91 (-20.15, 22.04) | - | -4.04 (-20.37, 12.39) | -4.17 (-24.64, 16.93) |
| HIFU+Oxytocin | 7.77 (1.2, 14.68) | -23.05 (-82.72, -4.63) | 4.99 (-11.19, 21.07) | 4.04 (-12.39, 20.37) | - | -0.1 (-15.9, 16.5) |
| HIFU+Microbubble Contrast Agent+Oxytocin | 7.89 (-6.92, 22.32) | -23.1 (-85.93, -2.62) | 5.07 (-16.07, 25.72) | 4.17 (-16.93, 24.64) | 0.1 (-16.5, 15.9) | - |

Table S4 League table of sonication energy

|  | HIFU | HIFU+GnRH-a | HIFU+Percutaneous Ethanol Injection | HIFU+Oxytocin |
| --- | --- | --- | --- | --- |
| HIFU | - | 22.24 (-34.18, 78.78) | **-233.05 (-286.68, -179.1)** | 6.27 (-69.27, 81.86) |
| HIFU+GnRH-a | -22.24 (-78.78, 34.18) | - | -255.5 (-333.29, -177.05) | -15.78 (-111.16, 78.29) |
| HIFU+Percutaneous Ethanol Injection | 233.05 (179.1, 286.68) | 255.5 (177.05, 333.29) | - | 239.32 (145.93, 331.99) |
| HIFU+Oxytocin | -6.27 (-81.86, 69.27) | 15.78 (-78.29, 111.16) | -239.32 (-331.99, -145.93) | - |

Table S5 League table of sonication time

|  | HIFU | HIFU+GnRH-a | HIFU+Testosterone Propionate | HIFU+Percutaneous Ethanol Injection | HIFU+Microbubble Contrast Agent | HIFU+Oxytocin | HIFU+Microbubble Contrast Agent+Oxytocin |
| --- | --- | --- | --- | --- | --- | --- | --- |
| HIFU | - | -272.51 (-566.14, -10.21) | 49.23 (-469.54, 567.31) | -263.96 (-801.29, 273.07) | -272 (-565.93, 24.5) | -127.06 (-393.03, 180.38) | **-670.64 (-1185.07, -151.45)** |
| HIFU+GnRH-a | 272.51 (10.21, 566.14) | - | 320.84 (-255, 923.62) | 8.38 (-577.7, 627.95) | 0.38 (-388.76, 423.29) | 147.07 (-221.75, 584.07) | -398.76 (-965.3, 203.12) |
| HIFU+Testosterone Propionate | -49.23 (-567.31, 469.54) | -320.84 (-923.62, 255) | - | -312.1 (-1060.4, 432.15) | -319.71 (-913.85, 279.01) | -177.18 (-748.43, 440.55) | -718.35 (-1448.77, 12.5) |
| HIFU+Percutaneous Ethanol Injection | 263.96 (-273.07, 801.29) | -8.38 (-627.95, 577.7) | 312.1 (-432.15, 1060.4) | - | -8.35 (-621.97, 609.67) | 136.66 (-449.82, 770.57) | -405.51 (-1160.63, 345.79) |
| HIFU+Microbubble Contrast Agent | 272 (-24.5, 565.93) | -0.38 (-423.29, 388.76) | 319.71 (-279.01, 913.85) | 8.35 (-609.67, 621.97) | - | 142.99 (-246.07, 576.97) | -398.89 (-992.14, 196.16) |
| HIFU+Oxytocin | 127.06 (-180.38, 393.03) | -147.07 (-584.07, 221.75) | 177.18 (-440.55, 748.43) | -136.66 (-770.57, 449.82) | -142.99 (-576.97, 246.07) | - | -541.82 (-1155.42, 25.14) |
| HIFU+Microbubble Contrast Agent+Oxytocin | 670.64 (151.45, 1185.07) | 398.76 (-203.12, 965.3) | 718.35 (-12.5, 1448.77) | 405.51 (-345.79, 1160.63) | 398.89 (-196.16, 992.14) | 541.82 (-25.14, 1155.42) | - |

Table S6 League table of treatment time

|  | HIFU | HIFU+GnRH-a | HIFU+Testosterone Propionate | HIFU+Percutaneous Ethanol Injection | HIFU+Microbubble Contrast Agent | HIFU+Oxytocin |
| --- | --- | --- | --- | --- | --- | --- |
| HIFU | - | -19.66 (-37.2, -2.71) | -2.46 (-35.86, 30.91) | **-44.02 (-79.57, -8.42)** | -33.86 (-57.8, -10.01) | -10.02 (-25.91, 7.18) |
| HIFU+GnRH-a | 19.66 (2.71, 37.2) | - | 17.21 (-20.27, 54.74) | -24.37 (-63.6, 15.7) | -14.18 (-43.64, 15.45) | 9.58 (-13.28, 34.36) |
| HIFU+Testosterone Propionate | 2.46 (-30.91, 35.86) | -17.21 (-54.74, 20.27) | - | -41.55 (-90.14, 7.25) | -31.45 (-72.5, 9.47) | -7.74 (-43.78, 30.21) |
| HIFU+Percutaneous Ethanol Injection | 44.02 (8.42, 79.57) | 24.37 (-15.7, 63.6) | 41.55 (-7.25, 90.14) | - | 10.24 (-32.93, 53.13) | 33.95 (-4.59, 74) |
| HIFU+Microbubble Contrast Agent | 33.86 (10.01, 57.8) | 14.18 (-15.45, 43.64) | 31.45 (-9.47, 72.5) | -10.24 (-53.13, 32.93) | - | 23.77 (-4.36, 53.6) |
| HIFU+Oxytocin | 10.02 (-7.18, 25.91) | -9.58 (-34.36, 13.28) | 7.74 (-30.21, 43.78) | -33.95 (-74, 4.59) | -23.77 (-53.6, 4.36) | - |

Table S7 League table of sonication time for ablating 1 mm^3^ of lesion

|  | HIFU | HIFU+GnRH-a | HIFU+Oxytocin |
| --- | --- | --- | --- |
| HIFU | - | -2.31 (-5.91, 1.63) | **-0.04 (-0.05, -0.04)** |
| HIFU+GnRH-a | 2.31 (-1.63, 5.91) | - | 2.27 (-1.68, 5.87) |
| HIFU+Oxytocin | 0.04 (0.04, 0.05) | -2.27 (-5.87, 1.68) | - |

Table S8 League table of grayscale changes in target area

|  | HIFU | HIFU+GnRH-a | HIFU+Testosterone Propionate |
| --- | --- | --- | --- |
| HIFU | - | 1.07 (0.93, 1.24) | **1.41 (1.07, 1.93)** |
| HIFU+GnRH-a | 0.93 (0.81, 1.07) | - | 1.32 (0.97, 1.85) |
| HIFU+Testosterone Propionate | 0.71 (0.52, 0.93) | 0.76 (0.54, 1.03) | - |

Table S9 League table of pain at treatment site

|  | HIFU | HIFU+GnRH-a | HIFU+Microbubble Contrast Agent | HIFU+Oxytocin |
| --- | --- | --- | --- | --- |
| HIFU | - | 1.03 (0.84, 1.28) | **0.66 (0.45, 0.9)** | 1.16 (1, 1.35) |
| HIFU+GnRH-a | 0.97 (0.78, 1.19) | - | 0.63 (0.41, 0.93) | 1.12 (0.87, 1.45) |
| HIFU+Microbubble Contrast Agent | 1.52 (1.11, 2.23) | 1.58 (1.08, 2.43) | - | 1.77 (1.24, 2.65) |
| HIFU+Oxytocin | 0.86 (0.74, 1) | 0.89 (0.69, 1.15) | 0.57 (0.38, 0.81) | - |

Table S10 League table of radiating pain

|  | HIFU | HIFU+GnRH-a | HIFU+Oxytocin |
| --- | --- | --- | --- |
| HIFU | - | 0.69 (0.27, 1.67) | **0.44 (0.18, 0.94)** |
| HIFU+GnRH-a | 1.45 (0.6, 3.77) | - | 0.63 (0.18, 2.16) |
| HIFU+Oxytocin | 2.28 (1.06, 5.47) | 1.58 (0.46, 5.46) | - |
